# Distinct phenotypic clusters associated with progressive pulmonary fibrosis in anti-synthetase syndrome-associated interstitial lung disease

**DOI:** 10.3389/fimmu.2026.1899365

**Published:** 2026-08-13

**Authors:** Zongshuai Gao, Yunxia Zhu

**Affiliations:** 1 Department of Transfusion Medicine, Shanghai Sixth People’s Hospital, Shanghai Jiao Tong University School of Medicine, Shanghai, China; 2 Department of Laboratory Medicine, Shanghai East Hospital, Tongji University School of Medicine, Shanghai, China

**Keywords:** anti-synthetase syndrome, disease trajectories, interstitial lung disease (ILD), progressive pulmonary fibrosis, unsupervised hierarchical cluster analysis

## Abstract

**Background:**

The clinical manifestations of anti-synthetase syndrome-associated interstitial lung disease (ASyS-ILD) are highly heterogeneous, and the mechanisms underlying its development and progression remain poorly understood. This study aimed to identify distinct clinical phenotypes of ASyS-ILD and clusters associated with progressive pulmonary fibrosis (PPF) using unsupervised cluster analysis.

**Methods:**

A total of 134 patients with ASyS-ILD were retrospectively enrolled. Unsupervised hierarchical clustering based on Gower distance and Ward’s method was performed. The optimal number of clusters was determined by silhouette analysis. Clinical manifestations, laboratory findings, pulmonary function, and prognosis were compared among clusters. A simplified decision tree model based on classification and regression tree (CART) analysis was further developed for cluster assignment.

**Results:**

Three distinct clinical clusters were identified. Cluster 1 (n=51, 38.1%) represented a relatively stable phenotype, characterized by mild systemic inflammation, preserved lung function, and favorable outcomes. Cluster 2 (n=49, 36.6%) exhibited dermatomyositis-like manifestations, including prominent muscle weakness (57.1%), Gottron’s sign (53.1%) and V sign rash (16.3%), and elevated muscle-associated enzymes. In addition, systemic inflammatory markers were significantly increased in cluster 2, including CRP (1.13 ng/mL), ESR (26.0 mm/h), ferritin (204.1 ng/mL). Cluster 3 (n=34, 25.4%) represented a PPF phenotype, characterized by significantly elevated fibrosis-related biomarkers, including CA125 (22.3 U/mL), CA15-3 (50.6 U/mL), and CYFRA21-1 (8.07 ng/mL), together with severe pulmonary involvement, markedly impaired DLCO (44.6%), and the highest prevalence of PPF (47.1%). Notably, Cluster 3 exhibited the highest all-cause mortality (23.5%), and Kaplan–Meier analysis further confirmed its worse prognosis among the three clusters (log-rank p=0.029). For rapid cluster classification, a simplified decision tree model incorporating CYFRA21-1, serum albumin, and neutrophil count was further developed and demonstrated good performance.

**Conclusions:**

ASyS-ILD comprises three distinct phenotypic subgroups with different clinical manifestations and outcomes. Cluster-based phenotyping may improve risk stratification and facilitate individualized management in patients with ASyS-ILD.

## Introduction

Anti-synthetase syndrome (ASyS) is an autoimmune disease characterized by the presence of anti-aminoacyl transfer RNA synthetase (anti-ARS) antibodies (1). Interstitial lung disease (ILD) is one of the most prominent manifestations of ASyS and is strongly associated with poor prognosis (2). However, ASyS-associated ILD (ASyS-ILD) exhibits marked clinical heterogeneity, ranging from predominantly inflammatory changes to fibrotic remodeling (3).

Previous studies have demonstrated that fibrosis may continue to progress in a subset of patients with ASyS-ILD, eventually leading to progressive pulmonary fibrosis (PPF) (4–9). However, by the time PPF is diagnosed, patients are often already at an advanced stage of disease, accompanied by worsening respiratory symptoms, progressive decline in lung function, and increased mortality (10). Therefore, early identification of patients at high risk for disease progression is of considerable clinical importance.

Several studies have attempted to identify predictors associated with PPF development in ASyS-ILD (4–6, 9). However, most previous analyses were based on conventional variable-centered approaches that evaluated individual clinical or laboratory factors separately. Given the substantial heterogeneity of ASyS-ILD, such approaches may not adequately capture the complex interactions among multidimensional disease features or identify clinically meaningful phenotypic subgroups.

Cluster analysis, an unsupervised machine learning approach, enables patient stratification by grouping individuals with similar clinical characteristics. It has been increasingly applied in heterogeneous diseases, such as autoimmune and respiratory diseases, to identify distinct phenotypes and improve risk stratification. Nevertheless, studies specifically exploring phenotype clustering in ASyS-ILD, particularly in relation to PPF risk and prognosis, remain limited.

Therefore, in the present study, we performed unsupervised hierarchical cluster analysis to identify distinct clinical phenotypes in patients with ASyS-ILD and investigate their associations with PPF development and long-term outcomes. In addition, we further developed a simplified decision tree model to facilitate rapid cluster classification in clinical practice.

## Materials and methods

### Study population

Inclusion criteria were as follows: (1) age ≥ 18 years; (2) a definite diagnosis of ASyS according to the Connors classification criteria (11); (3) ILD confirmed by chest high-resolution computed tomography (HRCT) based on thoracic radiologist reports. Exclusion criteria were as follows: (1) insufficient follow-up data to assess PPF development; and (2) ILD attributable to other causes, such as environmental exposures or drug-induced lung injury. Finally, A total of 134 ASyS-ILD patients from August 2017 to May 2025 in Shanghai East Hospital and Shanghai Sixth People’s Hospital were enrolled in this study.

### Data collection

Clinical, laboratory, imaging, and pulmonary function test (PFT) data were retrospectively collected from medical records of ASyS-ILD patients during the initial admission. ILD patterns were based on reports of chest HRCT by trained chest radiologists. Detection of anti-ARS antibodies at diagnosis was performed using a commercial line blot immunoassay kit (EUROLINE Autoimmune Inflammatory Myopathies 15 Ag, Lübeck, Germany). Survival status was obtained through medical records and telephone interviews. Follow-up was completed in May 2026, and patients were followed until death or the last available follow-up.

### Definition of the variables

According to the guideline (10), PPF was defined as at least two of the following three criteria occurring within the past year with no alternative explanation: (1) Worsening respiratory symptoms; (2) Physiological evidence of disease progression (either of the following): a. Absolute decline in FVC >5% predicted within 1 year of follow-up; b. Absolute decline in DLCO (corrected for Hb) >10% predicted within 1 year of follow-up; (3) Radiological evidence of disease progression (one or more of the following): a. Increased extent or severity of traction bronchiectasis and bronchiolectasis; b. New ground-glass opacity with traction bronchiectasis; c. New fine reticulation; d. Increased extent or increased coarseness of reticular abnormality; e. New or increased honeycombing; f. Increased lobar volume loss. Rapidly progressive ILD (RP-ILD) was defined as the presence of at least two of the following features occurring within 3 months: (1) progressive worsening of dyspnea; (2)rapid deterioration of interstitial abnormalities on HRCT; (3) >10% decrease in forced vital capacity (FVC) or >10mm Hg decrease in partial pressure of oxygen (PaO_2_) (12). The neutrophil-to-lymphocyte ratio (NLR) was calculated by dividing the absolute neutrophil count by the absolute lymphocyte count. Onset age was defined as the age at the first manifestation of ASyS.

### Ethical policy

This study was approved by the Ethics Committee of the Shanghai East Hospital (ethics approval information:2024YS-136). Due to the retrospective nature of the study and the anonymity of the data, the requirement for informed consent was waived.

### Cluster analysis

Hierarchical clustering analysis was performed to identify distinct clinical phenotypes. Based on clinical relevance, both continuous variables and categorical variables were included in the clustering model. Continuous variables were converted to numeric format and standardized using z-score normalization before clustering. Gower distance was used to calculate pairwise dissimilarities for mixed-type variables, followed by hierarchical clustering using Ward’s minimum variance method. The optimal number of clusters was determined using silhouette analysis. Cluster stability was evaluated using bootstrap resampling (1,000 iterations), and the mean Jaccard similarity coefficient was calculated for each cluster.

To improve clinical interpretability, a decision tree model was subsequently constructed using the Classification and Regression Tree (CART) algorithm. Cluster derived from hierarchical clustering was used as the dependent variable, and clustering variables were included as candidate predictors. Patients were randomly divided into a training cohort (70%) and a validation cohort (30%). The training cohort was used to construct the decision tree model, while the validation cohort was used for internal validation. Tree complexity was controlled by setting the complexity parameter (cp = 0.01), minimum split size (minsplit = 20), and maximum tree depth (maxdepth = 5). Model performance was evaluated using confusion matrices in both the training and validation cohorts.

### Statistical analysis

All statistical analyses were performed using R software (version 4.3.3; R Foundation for Statistical Computing, Vienna, Austria) within the RStudio environment. Continuous variables were presented as mean ± standard deviation (SD) or median (interquartile range [IQR]), as appropriate. Data normality was assessed using the Shapiro–Wilk test. For comparisons among the three clusters, one-way analysis of variance (ANOVA) was used for normally distributed variables and the Kruskal–Wallis test for non-normally distributed variables. Comparisons between two groups were performed using Student’s t test or the Mann–Whitney U test, as appropriate. Categorical variables were expressed as frequencies (percentages) and compared using the chi-square test or Fisher’s exact test. *Post hoc* pairwise comparisons were adjusted using Bonferroni correction when overall group differences were statistically significant. Kaplan–Meier survival analysis with the log-rank test was performed to compare survival outcomes among clusters.

## Results

### Baseline characteristics of the overall cohort

A total of 134 patients with ASyS-ILD were enrolled in this study, including 95 females (70.9%), with a mean onset age of 53.3 ± 10.3 years. During a median follow-up duration of 42.0 months, 34 patients (25.4%) fulfilled the criteria for PPF. Compared with non-PPF patients (**Supplementary Table 1**), those with PPF were more likely to be male (47.1% vs. 23.0%, p=0.014) and have a smoking history (41.2% vs. 15.0%, p=0.003). Regarding laboratory findings, patients with PPF exhibited significantly higher levels of LDH (310.5 vs. 242.0 U/L, p=0.014), ferritin (268.1 vs. 108.1 ng/mL, p=0.001), CA125 (21.84 vs. 14.28 U/mL, p=0.006), CA19-9 (18.52 vs. 10.10 U/mL, p=0.005), CA15-3 (28.91 vs. 21.90 U/mL, p=0.012), and CYFRA21-1 (5.36 vs. 3.78 ng/mL, p=0.005). Pulmonary function was also significantly impaired in the PPF group, as reflected by lower FVC (61.6% vs. 77.8%, p=0.019) and DLCO (46.6% vs. 59.7%, p=0.011). In addition, all-cause mortality was markedly higher among patients with PPF than those without PPF (32.4% vs. 5.0%, p<0.001). Kaplan–Meier survival analysis further demonstrated significantly poorer long-term survival in patients with PPF (**Supplementary Figure 1**, log-rank: p < 0.001).

### Three novel clusters of ASyS patients

**Figure 1:**
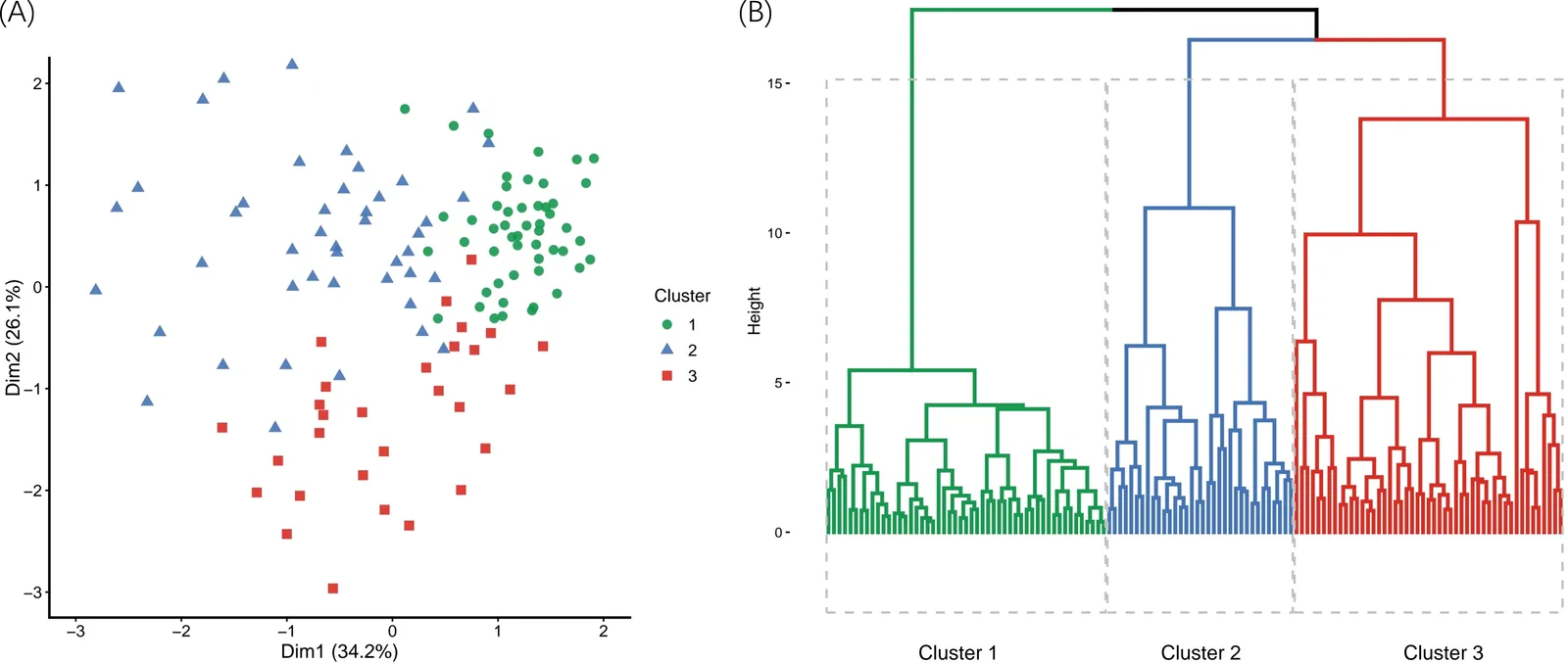
Visualization of clustering results in patients with ASyS-ILD. **(A)** Scatter plot map illustrating the clustering results of patients with ASyS-ILD. Each point represents an individual patient and is colored according to the corresponding cluster assignment; **(B)** Dendrogram showing the hierarchical clustering results and the distribution of patients among the three clusters identified by hierarchical cluster analysis. ASyS-ILD, anti-synthetase syndrome-associated interstitial lung disease.

Next, we investigated whether patients with ASyS-ILD could be classified into clinically and prognostically homogeneous subgroups. Unsupervised hierarchical clustering analysis based on clinical features and routine laboratory parameters was performed. Variables that are readily available and considered valuable for the diagnosis and disease assessment of ASyS were selected for the analysis (marked with a superscript “*” in **Table 1**). The silhouette analysis indicated that the optimal number of clusters was three (**Supplementary Figure 2**). As illustrated by the scatter plot map (**Figure 1A**) and cluster dendrogram (**Figure 1B**), patients were classified into three distinct clusters. The stability of the identified clusters was further assessed using bootstrap resampling (1,000 iterations). The mean Jaccard similarity coefficients for Clusters 1–3 were 0.854, 0.765, and 0.782, respectively, indicating satisfactory stability of the identified clusters.

**Table 1:** Baseline characteristics of three clusters of ASyS-ILD patients.

Variables	Total (n=134)	Cluster1 (n=51)	Cluster2 (n=49)	Cluster3 (n=34)	*P* value
Onset age, year*	53.26 (10.26)	52.14 (8.22)	52.02 (11.16)	56.74 (11.14)	0.072
Male, n (%)*	39 (29.1)	14 (27.5)	13 (26.5)	12 (35.3)	0.652
Duration from onset of symptoms to diagnosis, month	7.00 [3.00, 26.00]	7.00 [4.00, 24.00]	7.00 [3.00, 26.50]	8.00 [3.00, 28.50]	0.81
Ever smoking, n (%)*	29 (21.6)	7 (13.7)	11 (22.4)	11 (32.4)	0.122
Anti-synthetase antibodies					0.303
EJ, n (%)	38 (28.4)	14 (27.5)	13 (26.5)	11 (32.4)	
Jo1, n (%)	47 (35.1)	16 (31.4)	16 (32.7)	15 (44.1)	
PL12, n (%)	15 (11.2)	4 (7.8)	9 (18.4)	2 (5.9)	
PL7, n (%)	34 (25.4)	17 (33.3)	11 (22.4)	6 (17.6)	
Muscle weakness, n (%)*	49 (36.6)	12 (23.5)	28 (57.1)^ac^	9 (26.5)	0.001
Arthritis/arthralgia, n (%)*	56 (41.8)	23 (45.1)	21 (42.9)	12 (35.3)	0.656
Mechanic’s hands, n (%)*	79 (59.0)	29 (56.9)	29 (59.2)	21 (61.8)	0.903
Raynaud phenomenon, n (%)*	12 (9.0)	5 (9.8)	4 (8.2)	3 (8.8)	0.959
Heliotrope rash, n (%)*	21 (15.7)	6 (11.8)	11 (22.4)	4 (11.8)	0.261
Gottron’s sign, n (%)*	47 (35.1)	15 (29.4)	26 (53.1) ^ac^	6 (17.6)	0.002
V sign, n (%)	12 (9.0)	4 (7.8)	8 (16.3) ^c^	0 (0.0)	0.035
Shawl sign, n (%)	15 (11.2)	5 (9.8)	9 (18.4)	1 (2.9)	0.084
Periungual erythema, n (%)	6 (4.5)	3 (5.9)	2 (4.1)	1 (2.9)	0.802
Fever, n (%)	48 (35.8)	8 (15.7)^ab^	23 (46.9)	17 (50.0)	0.001
Dyspnea, n (%)	97 (72.4)	36 (70.6)	31 (63.3)	30 (88.2)^c^	0.041
CT, n (%)					0.174
NSIP	90 (67.2)	38 (74.5)	30 (61.2)	22 (64.7)	
NSIP+OP	30 (22.4)	9 (17.6)	14 (28.6)	7 (20.6)	
UIP	9 (6.7)	1 (2.0)	3 (6.1)	5 (14.7)	
Unspecified	5 (3.7)	3 (5.9)	2 (4.1)	0 (0.0)	
RP-ILD, n (%)	45 (33.6)	9 (17.6)	13 (26.5)	23 (67.6)^bc^	<0.001
PPF, n (%)	34 (25.4)	7 (13.7)	11 (22.4)	16 (47.1)^b^	0.002
Laboratory tests					
ALT, u/L	24.00 [14.00, 40.50]	17.00 [12.00, 27.50]	39.00 [16.00, 67.00] ^ac^	24.00 [13.50, 32.00]	<0.001
AST, u/L*	21.00 [16.00, 32.75]	1f7.00 [15.00, 22.50]	33.00 [21.00, 53.00] ^ac^	20.50 [17.00, 26.50]	<0.001
ALB, g/L*	37.15 [34.77, 39.73]	39.80 [38.10, 41.50]^b^	34.90 [32.10, 36.20] ^ac^	36.65 [34.62, 38.12]	<0.001
LDH, u/L*	257.00 [197.50, 344.25]	201.00 [177.00, 235.00]	349.00 [250.00, 457.00]^a^	278.00 [236.00, 323.75]^b^	<0.001
CK, u/L*	72.00 [40.25, 209.25]	62.00 [43.00, 98.00]	171.00 [48.00, 839.00] ^ac^	60.50 [26.25, 118.25]	0.001
CRP, ng/mL*	0.64 [0.25, 1.84]	0.32 [0.24, 0.60]	1.13 [0.48, 2.34] ^a^	1.31 [0.25, 2.32]^b^	<0.001
ESR, mm/h*	21.50 [8.00, 33.75]	13.00 [6.00, 22.50]	26.00 [17.00, 44.00] ^a^	23.00 [9.25, 33.00]	<0.001
Ferritin, ng/mL*	138.90 [66.20, 267.20]	70.70 [37.20, 187.75]	204.10 [108.05, 361.07 ^a^	124.30 [74.10, 271.90]	0.005
CA125, u/mL*	16.19 [10.87, 26.72]	12.80 [9.60, 21.56]	16.30 [10.88, 32.30]	22.30 [16.19, 42.55]^b^	0.001
CA19-9, u/mL*	10.60 [6.01, 19.44]	10.60 [6.39, 18.57]	9.36 [5.03, 16.62]	16.40 [7.05, 28.10]	0.171
CA15-3, u/mL*	22.80 [13.85, 35.40]	21.16 [13.92, 28.48]	17.88 [12.05, 25.74]	50.60 [30.30, 86.19]^bc^	<0.001
CYFRA21-1, ng/mL*	4.10 [3.10, 5.89]	3.58 [2.21, 4.64]	3.82 [3.00, 4.58]	8.07 [6.65, 9.62]^bc^	<0.001
HGB, g/L*	127.00 [117.00, 136.00]	131.00 [119.50, 139.50]	122.00 [113.00, 134.00] ^a^	128.50 [122.00, 136.25]	0.031
PLT, ×10^9^/L	235.00 [190.50, 301.00]	208.00 [168.50, 265.00]	265.00 [207.00, 338.00] ^a^	235.00 [192.50, 298.00]	0.014
WBC, ×10^9^/L	7.43 [5.76, 9.91]	6.37 [5.08, 7.43]	9.91 [7.37, 13.50] ^ac^	7.53 [5.93, 9.16]	<0.001
NEUT, ×10^9^/L*	4.98 [3.66, 7.94]	4.03 [2.96, 5.16]	7.95 [5.30, 9.70] ^ac^	4.77 [3.95, 6.43]^b^	<0.001
LYMP, ×10^9^/L*	1.31 [0.90, 1.94]	1.45 [1.13, 2.17]	1.17 [0.70, 1.71] ^a^	1.33 [1.08, 1.83]	0.015
NLR	3.71 [2.35, 6.57]	2.46 [1.64, 3.80]	6.56 [3.72, 10.60] ^ac^	3.56 [2.44, 5.30]^b^	<0.001
Pulmonary function tests					
FVC, %*	72.80 [60.90, 89.20]	77.90 [68.00, 96.82]	69.00 [59.55, 88.05] ^a^	65.90 [57.18, 81.18]^b^	0.014
FEV1, %	72.10 [60.20, 87.55]	77.05 [64.38, 88.20]	70.90 [59.45, 87.95]	70.50 [59.68, 81.88]	0.287
FEV1/FVC	90.63 [83.43, 99.33]	88.55 [80.94, 96.55]	93.65 [82.64, 101.60]	91.58 [84.63, 103.25]	0.189
DLCO, %*	56.60 [44.90, 69.70]	64.00 [56.30, 71.80]	50.35 [41.10, 63.28] ^a^	44.55 [35.67, 58.93]^b^	<0.001
Treatment naïve, n (%)	52 (38.8)	24 (47.1)	18 (36.7)	10 (29.4)	0.245
Treatment during this admission					
GC dose>1mg/kg/d, n (%)	13 (9.7)	2 (3.9)	2 (4.1)	9 (26.5)	0.001
Immunosuppressants					
CTX, n (%)	35 (26.1)	15 (29.4)	12 (24.5)	8 (23.5)	0.79
CNI, n (%)	33 (24.6)	8 (15.7)	20 (40.8)	5 (14.7)	0.004
MMF, n (%)	16 (11.9)	10 (19.6)	1 (2.0)	5 (14.7)	0.022
IVIg, n (%)	21 (15.7)	2 (3.9)	8 (16.3)	11 (32.4)	0.002
Rituximab, n (%)	2 (1.5)	0 (0.0)	2 (4.1)	0 (0.0)	0.172
Follow-up duration, month	42.04 [27.27, 57.73]	42.04 [30.77, 54.54]	43.22 [27.04, 59.77]	42.52 [26.02, 52.99]	0.951
All cause death, n (%)	16 (11.9)	2 (3.9)	6 (12.2)	8 (23.5)^b^	0.024

*Variables were selected for unsupervised hierarchical clustering analysis. ^a^Presents statistical differences between cluster 1 and cluster 2, ^b^ Presents statistical differences between cluster 1 and cluster 3 and ^c^ Presents statistical differences between cluster 2 and cluster 3.

ALB, albumin; ALT, alanine transaminase; AST, aspartate transaminase; ASyS-ILD, anti-synthetase syndrome-associated interstitial lung disease; CK, creatine kinase; CNI, calcineurin inhibitor; CRP, C-reactive protein; CTX, cyclophosphamide; DLCO, diffusing capacity for carbon monoxide; ESR, erythrocyte sedimentation rate; FEV1, forced expiratory volume in one second; FVC, forced vital capacity; GC, glucocorticoid; HGB, hemoglobin; ILD, interstitial lung disease; IVIg, intravenous immunoglobulins; LDH, lactate dehydrogenase; LYMP, lymphocytes; MMF, mycophenolate mofetil; NEUT, neutrophil cell; NLR, neutrophil-to-lymphocyte ratio; NSIP, nonspecific interstitial pneumonia; OP, organizing pneumonia; PLT, platelet; PPF, progressive pulmonary fibrosis; RP-ILD, rapidly progressive interstitial lung disease; UIP, usual interstitial pneumonia; WBC, white blood cell.

**Figure 2:**
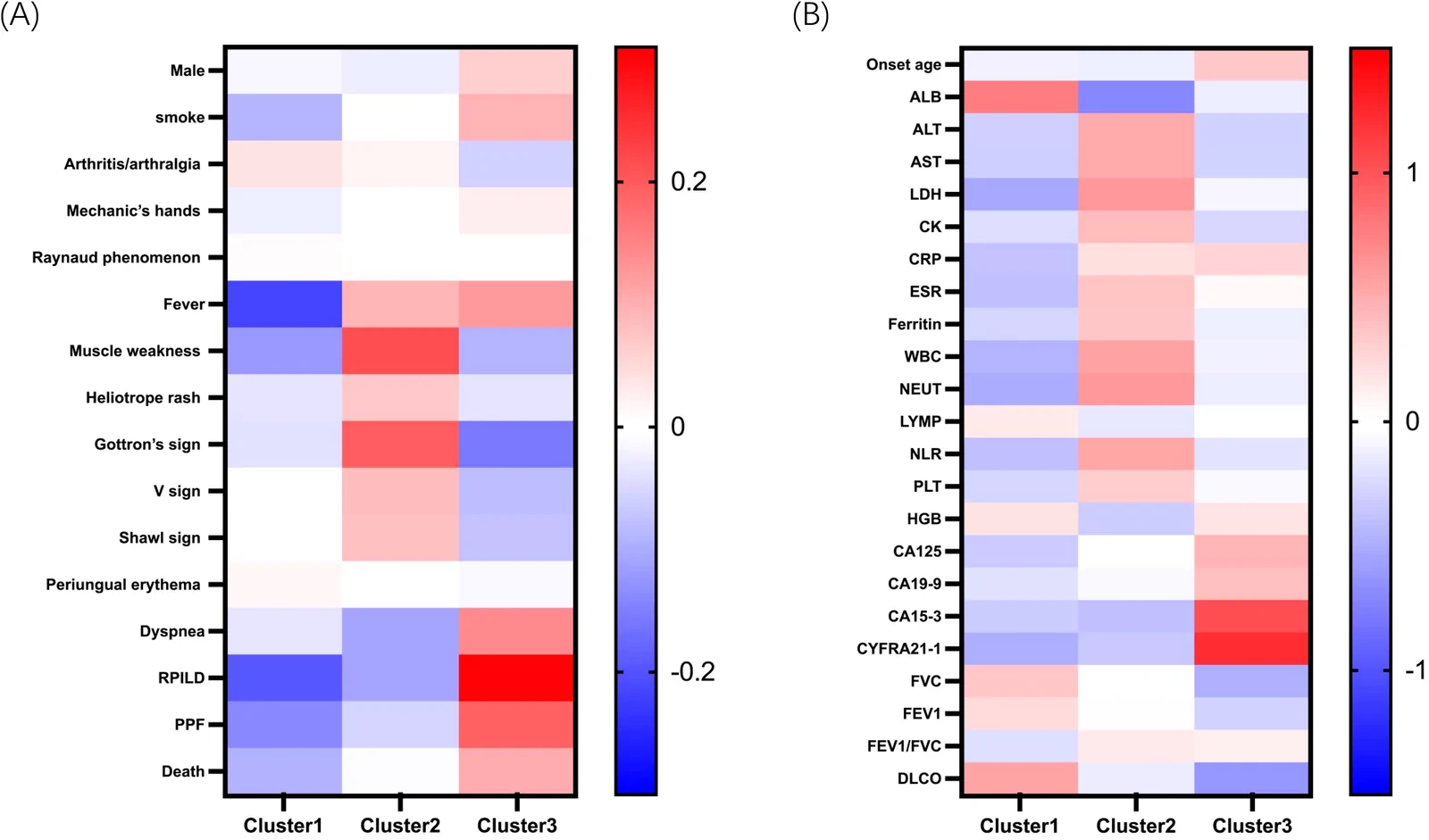
Heatmap of clinical characteristics across the three clusters. **(A)** Heatmap of standardized continuous variables across the three clusters; **(B)** Heatmap of standardized categorical variables across the three clusters. Continuous variables were standardized by Z-score transformation prior to visualization. Categorical variables were represented as centered occurrence proportions across clusters. Categorical variables were represented as centered occurrence proportions across clusters. Red indicates relative enrichment, and blue indicates relative depletion of a given feature compared with the overall cohort mean. ALB, albumin; ALT, alanine transaminase; AST, aspartate transaminase; CK, creatine kinase; CRP, C-reactive protein; DLCO, diffusing capacity for carbon monoxide; ESR, erythrocyte sedimentation rate; FEV1, forced expiratory volume in one second; FVC, forced vital capacity; HGB, hemoglobin; LDH, lactate dehydrogenase; LYMP, lymphocytes; NEUT, neutrophil cell; NLR, neutrophil-to-lymphocyte ratio; PLT, platelet; PPF, progressive pulmonary fibrosis; RPILD, rapidly progressive interstitial lung disease; WBC, white blood cell.

The baseline characteristics of the three clusters are summarized in **Table 1**, while the overall cluster profiles are presented in **Figures 2A, B**. Notably, the distribution of anti-ARS antibody specificities was comparable among the three clusters, with no significant differences observed (P = 0.303). Furthermore, the distribution of clusters was comparable between anti-Jo1 and non-Jo1 antibody groups (P = 0.191).

Specifically, Cluster 1 (n=51, 38.1%) represented a relatively stable phenotype, characterized by mild systemic inflammation, preserved lung function, and favorable clinical outcomes. Patients in this cluster showed the lowest frequency of fever (15.7% vs. 46.9% vs. 50.0%, cluster 1 vs. 2 vs. 3, p=0.001) among the three clusters. Laboratory findings further demonstrated a less inflammatory phenotype, with the highest serum albumin levels (39.8 vs. 34.9 vs. 36.7 g/L, cluster 1 vs. 2 vs. 3, p<0.001) and the lowest levels of C reactive protein (0.32 vs. 1.13 vs. 1.31 ng/mL, cluster 1 vs. 2 vs. 3, p<0.001), erythrocyte sedimentation rate (13.0 vs. 26.0 vs. 23.0 mm/hour, cluster 1 vs. 2 vs. 3, p<0.001), ferritin (70.7 vs. 204.1 vs. 124.3 ng/mL, cluster 1 vs. 2 vs. 3, p=0.005), neutrophil count (4.03 vs. 7.95 vs. 4.77 ×10^9/L, cluster 1 vs. 2 vs. 3, p<0.001), and neutrophil-to-lymphocyte ratio (2.46 vs. 6.56 vs. 3.56, cluster 1 vs. 2 vs. 3, p<0.001). Pulmonary function was relatively preserved in this cluster, as reflected by the highest forced vital capacity (77.9% vs. 69.0% vs. 65.9%, cluster 1 vs. 2 vs. 3, p=0.014) and diffusing capacity for carbon monoxide (64.0% vs. 50.4% vs. 44.6%, cluster 1 vs. 2 vs. 3, p<0.001). In addition, patients in cluster 1 had the lowest frequencies of RP-ILD (17.6% vs. 26.5% vs. 67.6%, cluster 1 vs. 2 vs. 3, p<0.001), PPF (13.7% vs. 22.4% vs. 47.1%, cluster 1 vs. 2 vs. 3, p=0.002), and all-cause mortality (3.9% vs. 12.2% vs. 23.5%, cluster 1 vs. 2 vs. 3, p=0.024), suggesting a comparatively favorable prognosis.

Cluster 2 (n=49, 36.6%) represented a dermatomyositis-like inflammatory phenotype. Muscle weakness was significantly more frequent in this cluster (23.5% vs. 57.1% vs. 26.5%, cluster 1 vs. 2 vs. 3, p=0.001), accompanied by a higher prevalence of Gottron’s sign (29.4% vs. 53.1% vs. 17.6%, cluster 1 vs. 2 vs. 3, p=0.002) and V sign rash (7.8% vs. 16.3% vs. 0%, cluster 1 vs. 2 vs. 3, p=0.035) compared with the other clusters. Consistently, patients in cluster 2 exhibited the highest levels of muscle-associated enzymes, including creatine kinase (62.0 vs. 171.0 vs. 60.5 U/L, cluster 1 vs. 2 vs. 3, p=0.001), lactate dehydrogenase (201.0 vs. 349.0 vs. 278.0 U/L, cluster 1 vs. 2 vs. 3, p<0.001), alanine aminotransferase (17.0 vs. 39.0 vs. 24.0 U/L, cluster 1 vs. 2 vs. 3, p<0.001), and aspartate aminotransferase (17.0 vs. 33.0 vs. 20.5 U/L, cluster 1 vs. 2 vs. 3, p<0.001). This cluster also showed the most pronounced systemic inflammatory profile, with elevated CRP (0.32 vs. 1.13 vs. 1.31 ng/mL, cluster 1 vs. 2 vs. 3, p<0.001), ESR (13.0 vs. 26.0 vs. 23.0 mm/hour, cluster 1 vs. 2 vs. 3, p<0.001), ferritin (70.7 vs. 204.1 vs. 124.3 ng/mL, cluster 1 vs. 2 vs. 3, p=0.005), white blood cell count (6.37 vs. 9.91 vs. 7.53 ×10^9/L, cluster 1 vs. 2 vs. 3, p<0.001), neutrophil count (4.03 vs. 7.95 vs. 4.77 ×10^9/L, cluster 1 vs. 2 vs. 3, p<0.001), and NLR (2.46 vs. 6.56 vs. 3.56, cluster 1 vs. 2 vs. 3, p<0.001). Fever was also common in cluster 2 (15.7% vs. 46.9% vs. 50.0%, cluster 1 vs. 2 vs. 3, p=0.001).

**Figure 3:**
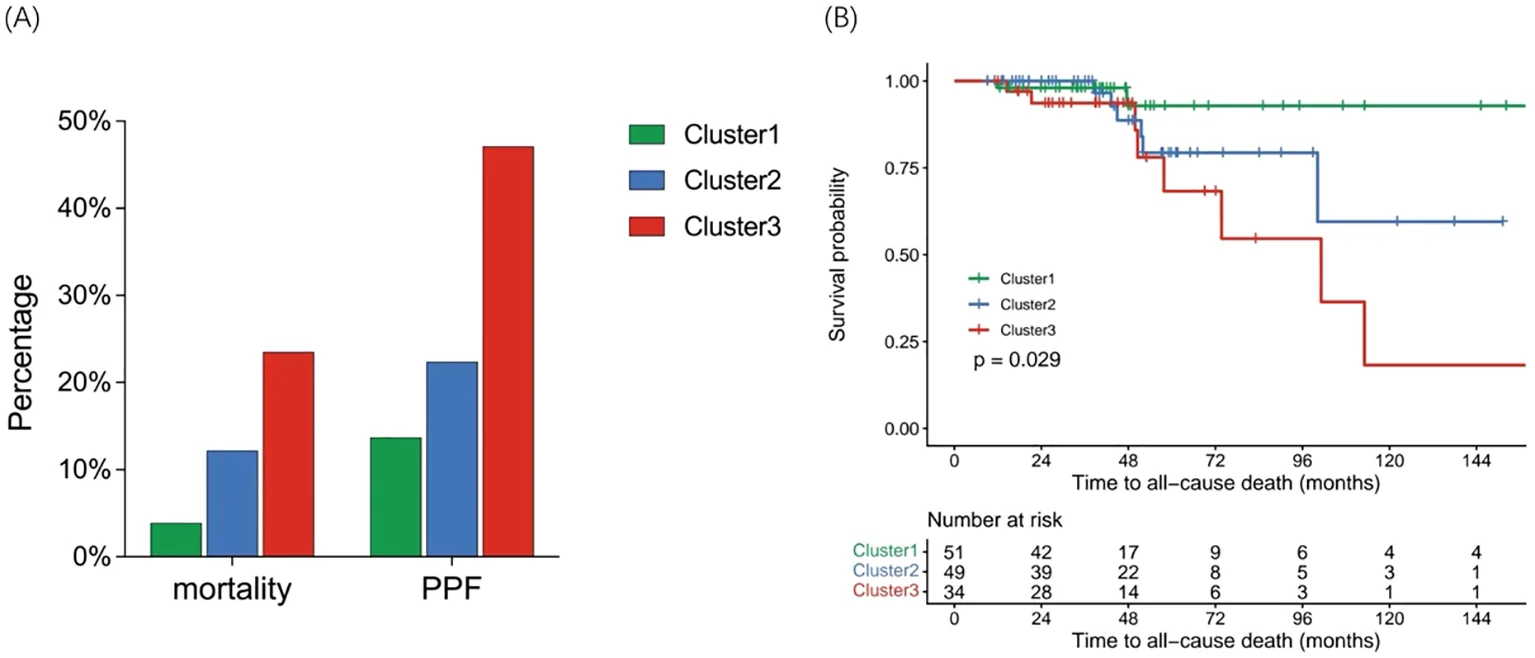
Clinical outcomes and survival analysis among the three clusters. **(A)** Comparison of the frequencies of progressive pulmonary fibrosis (PPF) and all-cause mortality among the three clusters; **(B)** Kaplan–Meier survival curves for all-cause mortality among patients with different clusters. The log-rank test was performed to compare survival probabilities among the three clusters.

Cluster 3 (n=34, 25.4%) represented a PPF cluster, characterized by severe pulmonary involvement and poor prognosis. Patients in this cluster exhibited significantly elevated serum biomarkers associated with lung injury and fibrosis, including CA125 (12.8 vs. 16.3 vs. 22.3 U/mL, cluster 1 vs. 2 vs. 3, p=0.001), CA15-3 (21.2 vs. 17.9 vs. 50.6 U/mL, cluster 1 vs. 2 vs. 3, p<0.001), and CYFRA21-1 (3.58 vs. 3.82 vs. 8.07 ng/mL, cluster 1 vs. 2 vs. 3, p<0.001), compared with the other two clusters. Clinically, dyspnea was highly prevalent in cluster 3 (70.6% vs. 63.3% vs. 88.2%, cluster 1 vs. 2 vs. 3, p=0.041). Pulmonary function was markedly impaired, with the lowest forced vital capacity (77.9% vs. 69.0% vs. 65.9%, cluster 1 vs. 2 vs. 3, p=0.014) and diffusing capacity for carbon monoxide (64.0% vs. 50.4% vs. 44.6%, cluster 1 vs. 2 vs. 3, p<0.001). In addition, patients in cluster 3 also showed the highest frequencies of RP-ILD (17.6% vs. 26.5% vs. 67.6%, cluster 1 vs. 2 vs. 3, p<0.001), PPF (13.7% vs. 22.4% vs. 47.1%, cluster 1 vs. 2 vs. 3, p=0.002, **Table 1** and **Figure 3A**), and all-cause mortality (3.9% vs. 12.2% vs. 23.5%, cluster 1 vs. 2 vs. 3, p=0.024).

### Clinical outcomes of three clusters

The cumulative all-cause mortality differed significantly among the three clusters, being highest in cluster 3 (23.5%), intermediate in cluster 2 (12.2%), and lowest in cluster 1 (3.9%) (**Table 1** and **Figure 3A**). As shown in **Figure 3B**, Kaplan–Meier survival analysis further demonstrated significant differences in long-term survival among the three clusters (log-rank p=0.029). Notably, the survival curves remained relatively close during the early follow-up period, whereas a clear separation of the survival curves emerged after approximately 48 months of follow-up. Cluster 1 consistently exhibited the most favorable survival probability throughout follow-up, while cluster 3 showed a marked decline in survival after 48 months, indicating a substantially worse long-term prognosis. Cluster 2 demonstrated an intermediate survival pattern between the other two clusters. These findings suggest that the cluster-based phenotypes were associated with distinct long-term clinical outcomes, particularly regarding late mortality risk. In the multivariable Cox regression analysis, after adjusting for baseline characteristics, lung function parameters, and treatment-related factors, cluster 2 (HR = 4.019, 95% CI 1.220–13.238, p=0.022) and cluster 3 (HR = 6.533, 95% CI 2.133–20.012, p=0.001) were independently associated with higher mortality risk compared with cluster 1 (**Supplementary Table 2**).

### Prediction of clusters by decision tree and internal validation.

**Figure 4:**
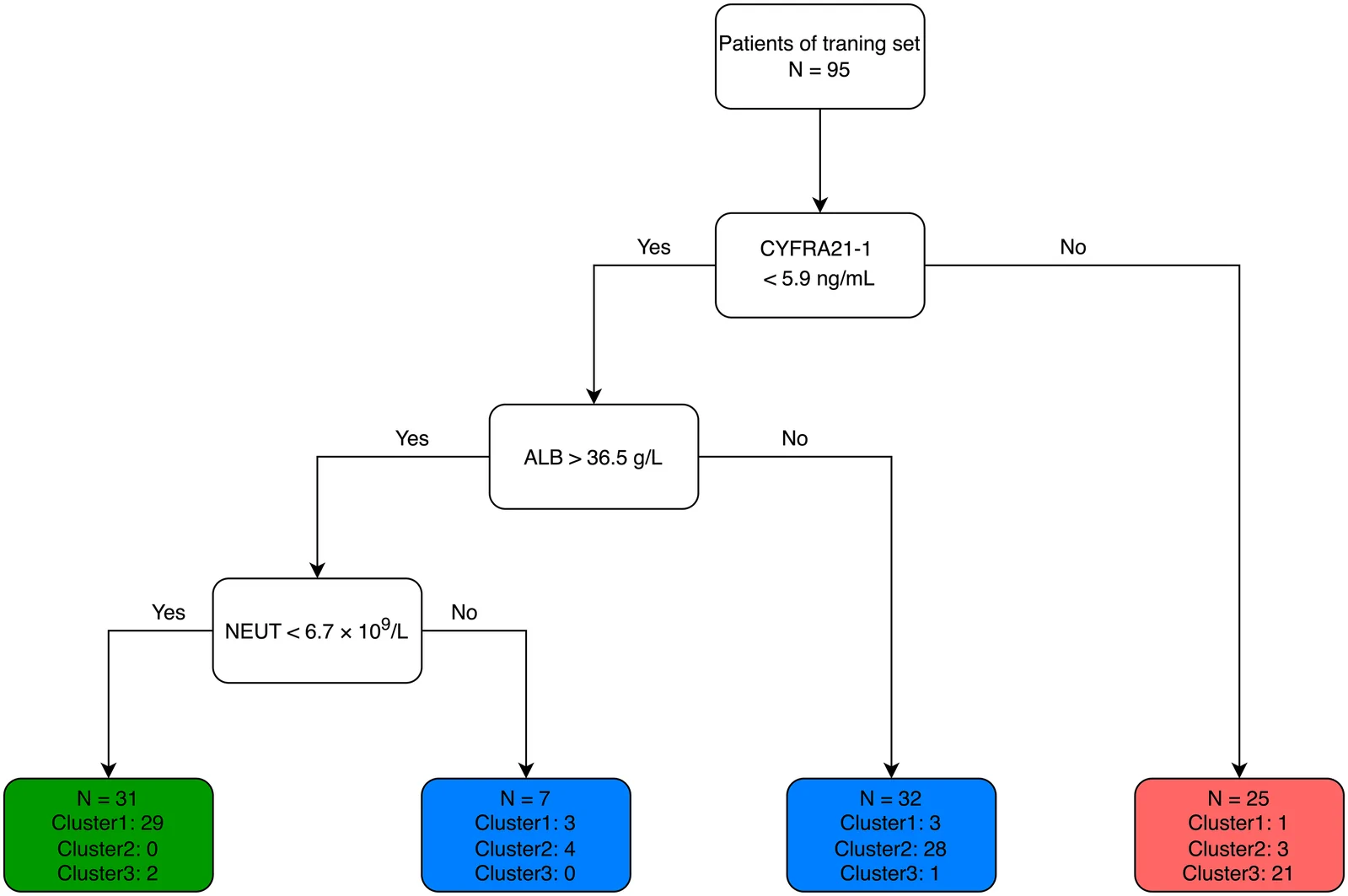
Simplified CART model for cluster classification. Classification and regression tree (CART) model for simplified cluster assignment in patients with ASyS-ILD. The final decision tree incorporated three laboratory variables, including CYFRA21-1, serum albumin (ALB), and neutrophil count (NEUT). Terminal nodes display the number of patients. Abbreviations: ASyS-ILD, anti-synthetase syndrome-associated interstitial lung disease.

To facilitate the classification of patients into distinct clinical clusters, we developed a simplified decision tree model using the CART algorithm. The entire cohort was randomly divided into a training set (n=95) and a validation set (n=39). Variables associated with cluster characteristics were initially included in the CART analysis, and the final simplified decision tree retained three readily accessible laboratory indicators: CYFRA21-1, serum albumin (ALB), and neutrophil count (NEUT) (**Figure 4**).

Specifically, patients with elevated CYFRA21–1 levels (≥5.9 ng/mL) were predominantly classified into cluster 3, with 21 of 25 patients (84.0%) belonging to this subgroup. Among patients with lower CYFRA21–1 levels (<5.9 ng/mL), those with preserved ALB levels (>36.5 g/L) and lower neutrophil counts (<6.7×10^9^/L) were mainly assigned to cluster 1, with 29 of 31 patients (93.5%) correctly classified. In contrast, patients with lower levels of CYFRA21-1 (<5.9 ng/mL) and ALB(≤36.5 g/L), but relatively elevated neutrophil counts (≥6.7×10^9^/L) were more likely to belong to cluster 2.

The CART model demonstrated good classification performance. In the training cohort, the overall prediction accuracy was 86.3% (κ=0.792), with balanced accuracies of 88.6%, 89.9%, and 90.9% for clusters 1, 2, and 3, respectively. In the validation cohort, the model maintained satisfactory predictive ability, achieving an overall accuracy of 82.1% (κ=0.727), with balanced accuracies of 89.2%, 83.3%, and 86.6% for clusters 1, 2, and 3, respectively. Detailed performance metrics, including the confusion matrix, sensitivity, specificity, positive predictive value, negative predictive value, balanced accuracy, and one-versus-rest AUC for each cluster in both the training and validation cohorts, are presented in **Supplementary Tables 3–6**. To further assess the stability of the model performance, we performed bootstrap resampling (1,000 iterations). The model showed stable classification performance, with a mean accuracy of 82.2% (95% CI, 69.2–94.9%), mean balanced accuracy of 86.5% (95% CI, 76.9–95.6%), mean κ of 0.725 (95% CI, 0.530–0.920), and mean macro-F1 score of 0.813 (95% CI, 0.676–0.939), consistent with the results observed in the validation cohort. These findings suggest that a simplified decision tree based on routine laboratory parameters may provide a practical approach for cluster assignment in clinical settings.

## Discussion

In the present study, we identified three clinically distinct phenotypic clusters in patients with ASyS-ILD using unsupervised hierarchical clustering analysis. These three clusters represented a clinically stable phenotype, a dermatomyositis-like inflammatory phenotype, and a PPF phenotype, respectively. These findings suggested the existence of clinically meaningful phenotypic subgroups with distinct disease trajectories and prognostic patterns in ASyS-ILD. Furthermore, for rapid cluster classification, we developed a simplified CART model based on three routinely available laboratory parameters.

Although several studies have explored phenotype clustering in ASyS (12–16), the relationship between cluster-defined phenotypes and PPF remains insufficiently investigated. To our knowledge, this is the first study to characterize phenotype clusters in relation to PPF development in patients with ASyS-ILD. Previous studies demonstrated that ARS antibodies are associated with distinct clinical manifestations (17–20). However, the associations between different anti-ARS antibody subtypes and pulmonary involvement or prognosis remain controversial. Non-Jo-1 antibodies have been associated with a fibrotic phenotype, increased risk of PPF, and poorer prognosis than those with anti-Jo-1 antibodies (4, 15, 21). Conversely, other studies did not identify significant differences in RP-ILD, PPF, or prognosis across different anti-ARS antibody subgroups (6, 12, 13). These inconsistencies suggest that antibody-defined classification alone may not adequately capture the multidimensional heterogeneity of ASyS-ILD. Therefore, an unsupervised clustering approach integrating diverse clinical and laboratory characteristics may provide more comprehensive disease stratification. In our study, antibody distribution was comparable across the three clusters, suggesting that clinical phenotypic clustering may capture additional heterogeneity beyond anti-ARS antibody classification.

Among the three identified clusters, cluster 3 may represent the most clinically important phenotype because of its close association with pulmonary involvement and poor long-term prognosis. Patients in this cluster showed markedly impaired pulmonary function, together with the highest prevalence of RP-ILD and PPF. In addition, serum biomarkers, including CA125, CA15-3, and CYFRA21-1, were significantly elevated in this cluster. Previous studies have identified these biomarkers as independent risk factors for PPF in ASyS-ILD patients (5, 22, 23). And these biomarkers are thought to reflect epithelial cell damage and pulmonary fibrosis in ILD (24–27). Therefore, the elevation of these markers in cluster 3 may reflect ongoing alveolar epithelial damage and progressive fibrotic remodeling. Notably, Kaplan–Meier analysis demonstrated that cluster 3 had the poorest prognosis among the three clusters, with survival differences becoming more apparent after approximately 48 months of follow-up. Given the markedly elevated fibrosis-related biomarkers and high prevalence of PPF in this subgroup, persistent fibrotic remodeling may partly contribute to its unfavorable long-term outcomes.

Cluster 2 was characterized by prominent dermatomyositis-like manifestations and marked systemic inflammatory activation. Patients in this subgroup exhibited significantly higher frequencies of muscle weakness, Gottron’s sign, and V sign rash, together with elevated muscle-associated enzymes. Similar clusters characterized by cutaneous manifestations and muscle involvement have also been reported in previous studies, supporting the reproducibility of our findings (12–14). Moreover, inflammatory markers such as CRP, ESR, ferritin, WBC count, NEUT count, and NLR were all highest in this cluster, indicating marked systemic inflammatory activation. Together with the prominent cutaneous and muscular manifestations, this subgroup may represent patients with a higher disease activity state. Interestingly, despite its prominent systemic inflammatory activity, cluster 2 exhibited a lower prevalence of PPF than cluster 3, suggesting divergent disease trajectories between these two subgroups. Consistent with their distinct clinical and biomarker profiles, cluster 2 may reflect a predominantly systemic inflammatory process, whereas cluster 3 appears more closely associated with progressive fibrotic remodeling.

In contrast, cluster 1 represented a relatively stable phenotype with favorable prognosis. Patients in this cluster exhibited the lowest levels of systemic inflammatory activity, relatively preserved pulmonary function, and the lowest prevalence of RP-ILD and PPF. These findings suggest that a subset of patients with ASyS-ILD may follow a relatively stable disease course with slower pulmonary progression. Consistent with our findings, a longitudinal study demonstrated that some patients with ASyS-ILD maintained relatively stable HRCT findings and pulmonary function during long-term follow-up (28). Early identification of such patients may help avoid overtreatment and facilitate individualized management strategies.

Another important finding of the present study was the development of a simplified decision tree model for cluster classification. Although hierarchical clustering analysis may provide valuable insights into disease phenotypes, its application in daily clinical practice is often limited by computational complexity and poor interpretability. To improve clinical applicability, we established a simplified decision tree model using only three parameters, including CYFRA21-1, ALB, and NEUT count. Despite its simplicity, the model demonstrated good predictive performance in both the training and validation cohorts. Of note, elevated CYFRA21–1 levels strongly identified patients belonging to the PPF cluster, further supporting the potential role of epithelial injury biomarkers in disease stratification. Using these routinely available laboratory parameters, clinicians may facilitate early identification of patients at high risk for progressive fibrotic disease, thereby enabling more intensive monitoring and timely optimization of therapeutic strategies. Collectively, this simplified model may provide a practical and interpretable tool for early phenotype recognition and risk stratification in ASyS-ILD.

Several limitations of this study should be acknowledged. First, this was a retrospective study conducted at two centers, which may introduce selection bias and unknown confounding bias. Second, the sample size was relatively limited, and the findings should therefore be interpreted cautiously. Third, external validation in independent cohorts was not performed, and the generalizability of the clustering model requires further confirmation. Fourth, more than 60% of patients had received immunosuppressive therapy before enrollment, which may have potentially affected the clinical features used for phenotype classification. Fifth, the number of patients remaining at risk decreased during long-term follow-up; therefore, survival estimates at later time points should be interpreted with caution. In addition, physical examination features included in the clustering analysis may be affected by documentation bias and inter-examiner variability. Finally, the underlying biological mechanisms responsible for the observed phenotypic heterogeneity remain unclear and warrant further investigation.

In conclusion, we identified three distinct clinical phenotypes in patients with ASyS-ILD using unsupervised hierarchical clustering analysis, which exhibited different clinical trajectories and prognostic patterns. Furthermore, we developed a simplified decision tree model based on routine laboratory indicators that may facilitate rapid phenotype classification in clinical practice. These findings highlight the substantial heterogeneity of ASyS-ILD and may facilitate improved risk stratification and individualized management.

## Data Availability

The data are available from the corresponding author upon reasonable request.
