# Supplementary material for: Distinct phenotypic clusters associated with progressive pulmonary fibrosis in anti-synthetase syndrome-associated interstitial lung disease
Source: Front Immunol. 2026 Aug 13;17:1899365. doi: 10.3389/fimmu.2026.1899365 (PMC13518561; doi:10.3389/fimmu.2026.1899365)
Supplement: Supplementary file 1 [file Table1.docx]

**Supplemental Table S1. Baseline characteristics of PPF and non-PPF patients.**

| **Variables** | **Total**  **(n=134)** | **non-PPF**  **(n=100)** | **PPF**  **(n=34)** | ***P* value** |
| --- | --- | --- | --- | --- |
| Onset age, year | 53.26 (10.26) | 53.44 (10.29) | 52.74 (10.30) | 0.731 |
| Male, n (%) | 39 (29.1) | 23 (23.0) | 16 (47.1) | 0.014 |
| Duration from onset of  symptoms to diagnosis, month | 7.00 [3.00, 26.00] | 7.00 [3.00, 24.50] | 7.00 [3.00, 36.00] | 0.964 |
| Ever smoking, n (%) | 29 (21.6) | 15 (15.0) | 14 (41.2) | 0.003 |
| Anti-synthetase antibodies |  |  |  | 0.054 |
| EJ, n (%) | 38 (28.4) | 27 (27.0) | 11 (32.4) |  |
| Jo1, n (%) | 47 (35.1) | 31 (31.0) | 16 (47.1) |  |
| PL12, n (%) | 15 (11.2) | 15 (15.0) | 0 (0.0) |  |
| PL7, n (%) | 34 (25.4) | 27 (27.0) | 7 (20.6) |  |
| Muscle weakness, n (%) | 49 (36.6) | 38 (38.0) | 11 (32.4) | 0.701 |
| Arthritis/arthralgia, n (%) | 56 (41.8) | 45 (45.0) | 11 (32.4) | 0.276 |
| Mechanic’s hands, n (%) | 79 (59.0) | 61 (61.0) | 18 (52.9) | 0.533 |
| Raynaud phenomenon, n (%) | 12 (9.0) | 9 (9.0) | 3 (8.8) | 1.000 |
| Heliotrope rash, n (%) | 21 (15.7) | 15 (15.0) | 6 (17.6) | 0.925 |
| Gottron’s sign, n (%) | 47 (35.1) | 38 (38.0) | 9 (26.5) | 0.313 |
| V sign, n (%) | 12 (9.0) | 10 (10.0) | 2 (5.9) | 0.705 |
| Shawl sign, n (%) | 15 (11.2) | 10 (10.0) | 5 (14.7) | 0.662 |
| Periungual erythema, n (%) | 6 (4.5) | 5 (5.0) | 1 (2.9) | 0.983 |
| Fever, n (%) | 48 (35.8) | 36 (36.0) | 12 (35.3) | 1.000 |
| Dyspnea, n (%) | 97 (72.4) | 72 (72.0) | 25 (73.5) | 1.000 |
| CT, n (%) |  |  |  | 0.566 |
| NSIP | 90 (67.2) | 67 (67.0) | 23 (67.6) |  |
| NSIP+OP | 30 (22.4) | 22 (22.0) | 8 (23.5) |  |
| UIP | 9 (6.7) | 6 (6.0) | 3 (8.8) |  |
| Unspecified | 5 (3.7) | 5 (5.0) | 0 (0.0) |  |
| RP-ILD, n (%) | 45 (33.6) | 30 (30.0) | 15 (44.1) | 0.195 |
| Laboratory tests |  |  |  |  |
| ALT, u/L | 24.00 [14.00, 40.50] | 23.00 [13.00, 39.75] | 31.50 [18.25, 40.50] | 0.210 |
| AST, u/L | 21.00 [16.00, 32.75] | 21.00 [16.00, 32.00] | 22.00 [17.00, 36.00] | 0.458 |
| ALB, g/L | 37.15 [34.77, 39.73] | 37.20 [34.90, 39.90] | 36.30 [33.70, 38.90] | 0.271 |
| LDH, u/L | 257.00 [197.50, 344.25] | 242.00 [196.50, 314.75] | 310.50 [238.75, 393.00] | 0.014 |
| CK, u/L | 72.00 [40.25, 209.25] | 65.00 [38.75, 216.25] | 81.50 [46.00, 186.75] | 0.505 |
| CRP, ng/mL | 0.64 [0.25, 1.84] | 0.56 [0.25, 1.69] | 0.97 [0.30, 2.17] | 0.199 |
| ESR, mm/h | 21.50 [8.00, 33.75] | 22.00 [8.75, 33.25] | 19.00 [7.25, 33.00] | 0.765 |
| Ferritin, ng/mL | 138.90 [66.20, 267.20] | 108.10 [65.60, 208.50] | 268.10 [135.70, 426.78] | 0.001 |
| CA125, u/mL | 16.19 [10.87, 26.72] | 14.28 [10.20, 24.83] | 21.84 [13.85, 43.80] | 0.006 |
| CA19-9, u/mL | 10.60 [6.01, 19.44] | 10.10 [5.79, 16.61] | 18.52 [6.98, 43.88] | 0.005 |
| CA15-3, u/mL | 22.80 [13.85, 35.40] | 21.90 [13.20, 32.60] | 28.91 [18.68, 67.52] | 0.012 |
| CYFRA21-1, ng/mL | 4.10 [3.10, 5.89] | 3.78 [2.90, 5.42] | 5.36 [3.67, 7.82] | 0.005 |
| HGB, g/L | 127.00 [117.00, 136.00] | 126.00 [115.75, 135.00] | 131.50 [126.25, 141.25] | 0.006 |
| PLT, ×10^9^/L | 235.00 [190.50, 301.00] | 236.00 [188.25, 302.75] | 221.00 [192.25, 298.00] | 0.563 |
| WBC, ×10^9^/L | 7.43 [5.76, 9.91] | 7.39 [5.54, 9.91] | 7.53 [6.26, 10.26] | 0.634 |
| NEUT, ×10^9^/L | 4.98 [3.66, 7.94] | 4.74 [3.61, 7.80] | 5.46 [3.92, 8.44] | 0.396 |
| LYMP, ×10^9^/L | 1.31 [0.90, 1.94] | 1.33 [1.00, 1.95] | 1.30 [0.82, 1.80] | 0.548 |
| NLR | 3.71 [2.35, 6.57] | 3.69 [2.14, 6.56] | 3.71 [3.02, 6.45] | 0.570 |
| Pulmonary function tests |  |  |  |  |
| FVC, % | 72.80 [60.90, 89.20] | 77.80 [63.20, 91.30] | 61.60 [59.38, 73.30] | 0.019 |
| FEV1, % | 72.10 [60.20, 87.55] | 77.00 [60.20, 88.15] | 65.25 [60.92, 76.47] | 0.053 |
| FEV1/FVC | 90.63 [83.43, 99.33] | 90.18 [83.29, 98.83] | 92.01 [83.58, 101.35] | 0.778 |
| DLCO, % | 56.60 [44.90, 69.70] | 59.70 [48.00, 70.60] | 46.55 [38.73, 57.35] | 0.011 |
| Treatment naïve, n (%) | 52 (38.8) | 44 (44.0) | 8 (23.5) | 0.056 |
| Treatment during this admission |  |  |  |  |
| GC dose>1mg/kg/d, n (%) | 13 (9.7) | 7 (7.0) | 6 (17.6) | 0.140 |
| Immunosuppressants |  |  |  |  |
| CTX, n (%) | 35 (26.1) | 29 (29.0) | 6 (17.6) | 0.282 |
| CNI, n (%) | 33 (24.6) | 23 (23.0) | 10 (29.4) | 0.604 |
| MMF, n (%) | 16 (11.9) | 11 (11.0) | 5 (14.7) | 0.787 |
| IVIg, n (%) | 21 (15.7) | 14 (14.0) | 7 (20.6) | 0.522 |
| Rituximab, n (%) | 2 (1.5) | 2 (2.0) | 0 (0.0) | 0.990 |
| Follow-up duration, month | 42.04 [27.27, 57.73] | 41.53 [26.78, 59.25] | 45.82 [29.54, 53.76] | 0.560 |
| All cause death, n (%) | 16 (11.9) | 5 (5.0) | 11 (32.4) | <0.001 |

Abbreviations: ALB, albumin; ALT, alanine transaminase; AST, aspartate transaminase; CK, creatine kinase; CNI, calcineurin inhibitor; CRP, C-reactive protein; CTX, cyclophosphamide; DLCO, diffusing capacity for carbon monoxide; ESR, erythrocyte sedimentation rate; FEV1, forced expiratory volume in one second; FVC, forced vital capacity; GC, glucocorticoid; HGB, hemoglobin; ILD, interstitial lung disease; IVIg, intravenous immunoglobulins; LDH, lactate dehydrogenase; LYMP, lymphocytes; MMF, mycophenolate mofetil; NEUT, neutrophil cell; NLR, neutrophil-to-lymphocyte ratio; NSIP, nonspecific interstitial pneumonia; OP, organizing pneumonia; PLT, platelet; PPF, progressive pulmonary fibrosis; RP-ILD, rapidly progressive interstitial lung disease; UIP, usual interstitial pneumonia; WBC, white blood cell.

**Supplemental Table S2. Multivariable Cox regression analysis for overall survival**

| Variables | Hazard Ratio (HR) | 95% CI | P value |
| --- | --- | --- | --- |
| Cluster |  |  | 0.004 |
| Cluster 1 | Reference |  |  |
| Cluster 2 | 4.019 | 1.220–13.238 | 0.022 |
| Cluster 3 | 6.533 | 2.133–20.012 | 0.001 |
| Onset age | 1.070 | 0.992–1.153 | 0.079 |
| Female | 4.230 | 0.292–61.316 | 0.290 |
| Ever smoking | 5.718 | 0.673–48.575 | 0.110 |
| RP-ILD | 0.481 | 0.083–2.799 | 0.415 |
| ALB | 0.863 | 0.681–1.092 | 0.220 |
| LDH | 0.999 | 0.992–1.007 | 0.854 |
| NLR | 0.864 | 0.686–1.088 | 0.215 |
| FVC | 1.041 | 0.956–1.134 | 0.351 |
| DLCO | 0.936 | 0.865–1.011 | 0.094 |
| GC dose>1mg/kg/d | 6.862 | 0.477–98.631 | 0.157 |
| CTX | 1.097 | 0.156–7.716 | 0.926 |
| CNI | 0.211 | 0.018–2.484 | 0.216 |
| MMF | 25.018 | 3.066–204.148 | 0.003 |
| IVIG | 0.086 | 0.006–1.229 | 0.071 |
| Rituximab | 0.004 | –* | 0.994 |

Abbreviations: ALB, albumin; CNI, calcineurin inhibitor; CTX, cyclophosphamide; DLCO, diffusing capacity for carbon monoxide; FVC, forced vital capacity; GC, glucocorticoid; IVIg, intravenous immunoglobulins; LDH, lactate dehydrogenase; MMF, mycophenolate mofetil; NLR, neutrophil-to-lymphocyte ratio; RP-ILD, rapidly progressive interstitial lung disease. The HR and 95% CI were not estimable because of the limited number of events.

**Supplemental Table S3. Confusion matrix for the CART model in the training cohort.**

|  | Actual Cluster 1 | Actual Cluster 2 | Actual Cluster 3 |
| --- | --- | --- | --- |
| Predicted Cluster 1 | 29 | 0 | 2 |
| Predicted Cluster 2 | 6 | 32 | 1 |
| Predicted Cluster 3 | 1 | 3 | 21 |

Abbreviations: CART, Classification and regression tree.

**Supplemental Table S4. Detailed performance metrics of the CART model in the training cohort.**

|  | Cluster 1 | Cluster 2 | Cluster 3 |
| --- | --- | --- | --- |
| Sensitivity | 0.806 | 0.914 | 0.875 |
| Specificity | 0.966 | 0.883 | 0.944 |
| Positive Predictive Value | 0.936 | 0.821 | 0.840 |
| Negative Predictive Value | 0.891 | 0.946 | 0.957 |
| Balanced Accuracy | 0.886 | 0.899 | 0.909 |
| One-versus-rest AUC | 0.930 | 0.937 | 0.925 |

Abbreviations: AUC, Area under the receiver operating characteristic curve; CART, Classification and regression tree.

**Supplemental Table S5. Confusion matrix for the CART model in the validation cohort.**

|  | Actual Cluster 1 | Actual Cluster 2 | Actual Cluster 3 |
| --- | --- | --- | --- |
| Predicted Cluster 1 | 13 | 1 | 1 |
| Predicted Cluster 2 | 2 | 11 | 1 |
| Predicted Cluster 3 | 0 | 2 | 8 |

Abbreviations: CART, Classification and regression tree.

**Supplemental Table S6. Detailed performance metrics of the CART model in the validation cohort.**

|  | Cluster 1 | Cluster 2 | Cluster 3 |
| --- | --- | --- | --- |
| Sensitivity | 0.867 | 0.786 | 0.800 |
| Specificity | 0.917 | 0.880 | 0.931 |
| Positive Predictive Value | 0.867 | 0.786 | 0.800 |
| Negative Predictive Value | 0.917 | 0.880 | 0.931 |
| Balanced Accuracy | 0.892 | 0.833 | 0.866 |
| One-versus-rest AUC | 0.928 | 0.864 | 0.871 |

Abbreviations: AUC, Area under the receiver operating characteristic curve; CART, Classification and regression tree.

Supplemental Figure S1. Kaplan–Meier survival curves for all-cause mortality of patients with and without progressive pulmonary fibrosis (PPF). The log-rank test was performed to compare survival probabilities.

Supplemental Figure S2. Determination of the optimal number of clusters

The optimal number of clusters was determined using average silhouette width analysis. Three clusters were ultimately identified as the optimal clustering solution for subsequent analyses.
